# Normal Weight Obesity Is Associated with Metabolic Syndrome and Insulin Resistance in Young Adults from a Middle-Income Country

**DOI:** 10.1371/journal.pone.0060673

**Published:** 2013-03-28

**Authors:** Francilene B. Madeira, Antônio A. Silva, Helma F. Veloso, Marcelo Z. Goldani, Gilberto Kac, Viviane C. Cardoso, Heloisa Bettiol, Marco A. Barbieri

**Affiliations:** 1 Physical Education Undergraduate Course, State University of Piauí, Teresina, Brazil; 2 Department of Public Health, Federal University of Maranhão, São Luís, Brazil; 3 Department of Pediatrics and Puericulture, Faculty of Medicine, Federal University of Rio Grande do Sul, Porto Alegre, Brazil; 4 Department of Social and Applied Nutrition, Josué de Castro Nutrition Institute, Federal University of Rio de Janeiro, Rio de Janeiro, Brazil; 5 Department of Puericulture and Pediatrics, Faculty of Medicine of Ribeirão Preto, University of São Paulo, Ribeirão Preto, Brazil; University of São Paulo, Brazil

## Abstract

**Objective:**

This population-based birth cohort study examined whether normal weight obesity is associated with metabolic disorders in young adults in a middle-income country undergoing rapid nutrition transition.

**Design and Methods:**

The sample involved 1,222 males and females from the 1978/79 Ribeirão Preto birth cohort, Brazil, aged 23–25 years. NWO was defined as body mass index (BMI) within the normal range (18.5–24.9 kg/m^2^) and the sum of subscapular and triceps skinfolds above the sex-specific 90th percentiles of the study sample. It was also defined as normal BMI and % BF (body fat) >23% in men and >30% in women. Insulin resistance (IR), insulin sensitivity and secretion were based on the Homeostasis Model Assessment (HOMA) model.

**Results:**

In logistic models, after adjusting for age, sex and skin colour, NWO was significantly associated with Metabolic Syndrome (MS) according to the Joint Interim Statement (JIS) definition (Odds Ratio OR = 6.83; 95% Confidence Interval CI 2.84–16.47). NWO was also associated with HOMA2-IR (OR = 3.81; 95%CI 1.57–9.28), low insulin sensitivity (OR = 3.89; 95%CI 2.39–6.33), and high insulin secretion (OR = 2.17; 95%CI 1.24–3.80). Significant associations between NWO and some components of the MS were also detected: high waist circumference (OR = 8.46; 95%CI 5.09–14.04), low High Density Lipoprotein cholesterol (OR = 1.65; 95%CI 1.11–2.47) and high triglyceride levels (OR = 1.93; 95%CI 1.02–3.64). Most estimates changed little after further adjustment for early and adult life variables.

**Conclusions:**

NWO was associated with MS and IR, suggesting that clinical assessment of excess body fat in normal-BMI individuals should begin early in life even in middle-income countries.

## Introduction

The prevalence of obesity (Body Mass Index - BMI ≥30 kg/m^2^) has increased worldwide over the past decades [1], [2], although more recent data suggest a slowing or levelling off of this trend [3]. In Brazil, from 1974–1975 to 2008–2009, the prevalence rates of obesity increased more than fourfold among men (2.8% to 12.4%) and more than twofold among women (from 8.0% to 16.9%) [4].

Obesity, defined as excess body fat (BF) [5], [6], has been evaluated in both clinical and epidemiological studies, using predominantly BMI [7], [8]. Studies have shown an association between extreme values of BMI and increased mortality [9], [10]. However, because BMI does not differentiate lean from fat mass, this indicator has limited accuracy for diagnosing individuals with excess BF presenting BMI within the normal range [11]–[13].

In the early eighties, Ruderman et al. described a specific type of obesity defined as metabolically obese normal weight subjects (MONW). These individuals were characterized by normal body weight and BMI, but presented hyperinsulinemia, insulin-resistance, and increased type 2 diabetes, hypertriglyceridemia and cardiovascular diseases predisposition [14], [15]. Few years later, De Lorenzo et al. [16] among other authors [17], [18] used the term normal weight obesity (NWO) to identify individuals who have normal body weight and BMI but high % BF, accompanied by total lean mass deficiency. Therefore, MONW is a subset of NWO and from a conceptual and clinical perspective it is important to differentiate these two conditions.

Some other studies have reported associations between NWO and metabolic disorders [16], [18]–[27]. In a study of the US population, individuals aged >20 years with NWO were four times more likely to develop metabolic syndrome (MS) than those with normal BMI and normal BF [18]. In another study carried out in Switzerland, which included only females of Caucasian origin aged 35–75 years, women with NWO had a higher cardiometabolic risk and higher prevalences of low high-density lipoprotein (HDL) cholesterol, high waist circumference (WC), high triglycerides and hyperglycaemia but a similar prevalence of hypertension compared to lean women [20].

However, to our knowledge, there are few studies reporting an association between NWO and metabolic disorders exclusively in young adults or coming from low and middle-income countries [28]–[30]. Studies in young populations are important because if NWO is associated with metabolic imbalances at an early age, clinical evaluation should change and preventive public policy actions should be redrawn and begin earlier in order to limit complications, as NWO individuals get older [6], [15], [31].

The objective of the present study was to evaluate the association between NWO and MS and insulin resistance (IR) in young adults from a population-based birth cohort performed in a middle-income country undergoing rapid nutrition transition, with adjustment for several early and adult life variables.

## Methods

### Ethics Statement

The project was approved by the Research Ethics Committee of the Clinics Hospital, Faculty of Medicine of Ribeirão Preto, University of São Paulo, Brazil. All participants signed an informed consent form.

### Study design and participants

Data were abstracted from the first Ribeirão Preto birth cohort study, Brazil, which started in 1978/79. Data were obtained at birth and at young adult age (23–25 years) [32].

A total of 9067 liveborn infants, delivered at the eight maternity hospitals of Ribeirão Preto, from June 1st 1978 to May 31, 1979 (corresponding to 98% of all live births), participated in this study. Losses due to refusal or early discharge amounted to 3.5%. All infants whose families did not reside in the city (2094) and twins (146) were excluded, leaving a total of 6827 live births [33].

From the original cohort of 6827 singleton liveborns, 343 participants were found to be deceased and 819 could not be traced, leaving 5665 singletons. One in three subjects belonging to the same geographic area was invited for medical examination. The first of every three names was selected from a list sorted by birth date in each geographic region and, if unavailable, the next name down was selected. In this traced group, losses to follow-up (N = 705) occurred because of refusal to participate, imprisonment, death after 20 years of age, or failure to attend the interview. Losses were replaced using the same sampling frame, resulting in 2,063 young adults (1,068 females) [32]. For this study, only subjects with normal BMI (18.5 to 24.9 kg/m^2^)[6] were included, comprising a total of 1,222 young adults. Details of the methodology have been published elsewhere [32], [33].

### Variables and data collection

At the time of their children's birth, the mothers answered a standardized questionnaire. Maternal schooling (≤4, 5 to 8, 9 to 11, ≥12 years), parity (1, 2 to 4, ≥5), type of delivery (vaginal, caesarean) and maternal smoking during pregnancy (yes, regardless of the number of cigarettes smoked, and no) were abstracted from this questionnaire. Birth weight was measured within 30 minutes of birth. Newborns were weighed naked on weekly calibrated mechanical scales with 10-g precision (Filizola, São Paulo, Brazil). Gestational age at birth in complete weeks was derived from the last normal menstrual period reported by the mother.

Participants answered a questionnaire containing information on socioeconomic, demographic and behavioural variables, and underwent physical examination when they were 23–25 years of age. The variables collected in adulthood were: age, sex, self-reported skin colour (classified as white/non-white), family income measured in minimum wages and classified into three categories (<5, 5 to 9.9 and ≥10), schooling in years (≤8, 9 to 11 and ≥12), marital status (single, cohabiting), smoking (yes, regardless of the number of cigarettes smoked and no), alcohol consumption in grams per day (none, ≤31 and >31), percentage of fat in the diet (measured with a food-frequency questionnaire and derived from equations using the Dietsys software, version 4.0 (National Cancer Institute, Bethesda, MD, USA) [34] and physical activity (sedentary, sufficiently active and active), according to the International Physical Activity Questionnaire (IPAQ) guidelines [35], [36]. A missing category was added to the family income variable because 91 participants did not report their income. Anthropometric measurements were taken by physicians or trained nurses with individuals wearing light clothing and no shoes, using a standard protocol [37]. Weight was measured to the nearest 100 g using mechanical scales (Filizola, São Paulo, Brazil). Height was measured to the nearest 0.1 cm using a freestanding wood stadiometer (University of São Paulo, Ribeirão Preto, Brazil). BMI was calculated as weight in kilograms divided by height in meters squared (kg/m^2^). A D-loop non-stretch fiberglass tape was used for WC and hip circumference (HC) measures. WC was measured as the smallest circumference between the ribs and the iliac crest while the participant was standing with the abdomen relaxed, at the end of a normal expiration. Where there was no natural waistline, the measurement was taken at the level of the umbilicus. HC was measured at the maximum circumference between the iliac crest and the crotch while the participant was standing. The triceps and subscapular skinfolds were measured with the Lange adipometer (Beta Technology, Santa Cruz, CA, USA), following Lohman's protocol [37]. Acceptable inter- and intra-observer agreement was achieved. For blood pressure measurements we used an Omron digital sphygmomanometer model 740 (Omron Healthcare, Lake Forest, IL, US), with 15-minute intervals between measurements, with the participants seated. This procedure was performed three times and the average of the last two measurements was used.

A 40 ml blood sample was collected from the subject after a 12 hour fast by a trained technician. Fasting blood glucose was measured by the GOD/PAP human diagnostic enzymatic calorimetric method (Chronolab AG, Zug, Germany) with a coefficient of variation of 4.2%. Low density lipoprotein (LDL) cholesterol, HDL-cholesterol and triglycerides were determined by an enzymatic calorimetric method using the Dade Behring XPand apparatus (Dade Behring, Liederbach, Germany) and reagents of Dade Behring Dimension clinical chemistry. Fasting insulin was measured by radioimmunoassay (insulin kit, DPC, Los Angeles, CA, USA) with a coefficient of variation of 7.9% [38].

MS was defined according to the Joint Interim Statement (JIS) of the IDF Task Force on Epidemiology and Prevention, National Heart, Lung and Blood Institute, American Heart Association, World Heart Federation, International Atherosclerosis Society and International Association for the Study of Obesity. The JIS criterion requires the presence of any three of the following: 1) central obesity (WC ≥90 cm for men and ≥80 cm for women, cut-off points used for South American populations); 2) increased triglycerides ≥150 mg/dL, use of lipid medications or self-reported diagnosis of hypertriglyceridemia; 3) low HDL-cholesterol (<40 mg/dL for men and <50 mg/dL for women); 4) increased blood pressure (BP) (systolic pressure ≥130 mmHg and/or diastolic pressure ≥85 mmHg, current usage of antihypertensive drugs or previous diagnosis of hypertension); and 5) high fasting blood glucose (≥100 mg/dL), current use of anti-diabetic medication or previously diagnosed diabetes [39].

IR was evaluated by the Homeostasis Model Assessment index [40]. HOMA2 insulin resistance, HOMA2 insulin sensitivity (the opposite of insulin resistance) and insulin secretor activity (HOMA2 β cell function) were determined using the HOMA2 computer model, which uses correctly solved nonlinear solutions (available from http://www.dtu.ox.ac.uk/index.php?maindocZ/homa/) and takes into account variations in hepatic and peripheral glucose resistance, increases in the insulin secretion curve for plasma glucose concentrations above 10 mmol/L (180 mg/dL) and the contribution of circulating proinsulin [41]. The cut-off proposed by the Brazilian Metabolic Syndrome Study (BRAMS) were used for the diagnosis of HOMA2-IR (>1.8) [42]. Since there were no cut-offs described for the Brazilian population, HOMA2 insulin sensitivity was considered to be low when <10^th^ percentile and HOMA2 β cell function was considered to be high if >90^th^ percentile of the study sample distribution.

The subjects whose BMI was 18.5 to 24.9 kg/m^2^ and whose sum of triceps and subscapular skinfolds was >90^th^ percentile of the study sample for each sex were classified as NWO, corresponding to >23.1% BF in men and >33.3% BF in women. We also defined NWO as a normal BMI and % BF >23% in men and >30% in women, using Slaughter's equations (derived for adolescents 8–18 years) from the sum of triceps and subscapular skinfolds [43].

### Statistical analysis

Statistical analysis was performed using the statistical package Stata version 12.0. Mean ± standard deviation or the 1st quartile, median and the 3rd quartile were presented when appropriate. Differences in mean values of demographic, dietetic, anthropometric and metabolic parameters according to the presence or absence of NWO were tested by the Student t-test when variables had a normal distribution or by the Mann-Whitney non-parametric test otherwise. Statistical differences in categorical variables according to NWO were evaluated using the chi-square test. Subsequently, we fitted logistic regression models, using NWO as the explanatory variable and separate models for each response variable - MS, its components (high WC, low HDL-cholesterol, high triglycerides, high blood pressure and high blood glucose), insulin resistance, insulin sensitivity, and β cell function. Three sequential models were presented: model 1 (adjusted for age, sex and skin colour), model 2 (adjusted for age, sex, skin colour and early life variables - birth weight, gestational age at birth, maternal schooling, parity, type of delivery and maternal smoking during pregnancy) and model 3 (adjusted for age, sex, skin colour, early and adult life variables (alcohol consumption, family income, schooling, marital status, smoking, percentage of fat in the diet and physical activity). Additional models were also further adjusted for WC to verify if associations between NWO and low HDL-cholesterol, high triglycerides, high blood pressure, high blood glucose, insulin resistance, low insulin sensitivity and high insulin secretion were independent from measures of central obesity. The models were not stratified by sex because no significant interactions between NWO and sex on MS or IR were detected. Odds ratios (OR) and their 95% confidence intervals (CI) were estimated.

## Results

The prevalence of MS according to the JIS definition was 3.1% (95%CI 1.8%–4.9%) for males and 0.9% (95%CI 0.3%–1.9%) for females. The prevalence of HOMA2-IR was 2.0% (95%CI 1.0%–3.6%) for men and 2.6% (95%CI 1.5%–4.1%) for women. The prevalence of MS was higher for males than for females (3.1% vs. 0.9%, P = 0.004). Low HDL-cholesterol was significantly higher among women (38.0%) than men (31.2%), whereas high blood pressure was much higher among men (28.9%) than women (3.3%). High blood glucose was also higher among men (4.4%) than women (1.7%). IR, high WC and triglycerides did not differ by sex (Table 1).

**Table 1:** Prevalences of metabolic syndrome, its components and insulin resistance by sex among young adults with body mass index within the normal range, 1978/79 Ribeirão Preto birth cohort.

Variables	Males (n = 546)		Females (n = 676)		P ^a^
	n	%	n	%	
**Metabolic Syndrome – JIS^b^**					0.004
No	529	96.9	670	99.1	
Yes	17	3.1	6	0.9	
**Waist circumference^c^**					0.852
Normal	509	93.2	632	93.5	
High	37	6.8	44	6.5	
**HDL-cholesterol^d^**					0.014
Normal	373	68.8	413	62.0	
Low	169	31.2	253	38.0	
**Triglycerides^e^**					0.439
Normal	509	93.9	618	92.8	
High	33	6.1	48	7.2	
**Blood pressure^f^**					<0.001
Normal	388	71.1	654	96.8	
High	158	28.9	22	3.3	
**Blood Glucose^g^**					0.004
Normal	520	95.6	656	98.4	
High	24	4.4	11	1.7	
**HOMA2-IR^h^**					0.539
≤1.8	528	98.0	642	97.4	
>1.8	11	2.0	17	2.6	

Abbreviations: HDL, High Density Lipoprotein; HOMA, Homeostasis Model Assessment; IR, Insulin Resistance. ^a^P value calculated by the chi-square test. ^b^defined according to the Joint Interim Statement (JIS) of the IDF Task Force on Epidemiology and Prevention, National Heart, Lung and Blood Institute, American Heart Association, World Heart Federation, International Atherosclerosis Society and International Association for the Study of Obesity. ^c^waist circumference (≥90 cm for men and ≥80 cm for women). ^d^increased triglycerides (≥150 mg/dL, use of lipid medications or self-reported diagnosis of hypertriglyceridemia). ^e^low HDL-cholesterol (<40 mg/dL for men and <50 mg/dL for women). ^f^increased blood pressure (BP) (systolic pressure ≥130 mmHg and/or diastolic pressure ≥85 mmHg, current usage of antihypertensive drugs or previous diagnosis of hypertension). ^g^high fasting blood glucose (≥100 mg/dL), current use of anti-diabetic medication or previously diagnosed diabetes. ^h^Cut-off point based on the Brazilian Metabolic Syndrome Study - BRAMS criterion (2009). Numbers may not add up to total because of missing values.

Subjects with NWO did not differ from those without NWO according to early life variables (Table 2). Subjects with NWO did not differ by sex, age, family income, schooling, marital status, smoking or alcohol consumption compared to those without NWO. Individuals of white skin colour (10.5% vs. 6.2%, P = 0.013) and with a sedentary life style (10.8% vs. 5.3%, P = 0.010) presented a higher prevalence of NWO than their counterparts. Mean percentage of fat in the diet was higher among NWO subjects than among their peers without NWO (Table 3).

**Table 2:** Normal weight obesity according to early life variables, 1978/79 Ribeirão Preto birth cohort.

Variables	Normal weight obesity^a^		P
	No (n = 1111) n (%)	Yes (n = 111) n (%)	
Maternal schooling (years)			0.786^b^
≤4	484 (91.7)	44 (8.3)	
5 to 8	284 (90.5)	30 (9.5)	
9 to 11	189 (90.9)	19 (9.1)	
≥12	130 (89.0)	16 (11.0)	
Maternal parity			0.101^b^
1	405 (88.6)	52 (11.4)	
2 to 4	595 (92.3)	50 (7.8)	
≥5	87 (92.5)	7 (7.5)	
Type of delivery			0.521^b^
Vaginal	768 (90.6)	80 (9.4)	
Cesarean	343 (91.7)	31 (8.3)	
Maternal smoking during pregnancy			0.384^b^
No	819 (91.3)	78 (8.7)	
Yes	268 (89.6)	31 (10.4)	
Birth weight (grams)	3238±500^c^	3299±436^c^	0.220^d^
Gestational age at birth (weeks)	39.0±1.8^c^	39.3±1.3^c^	0.071^d^

a Normal weight obesity defined as a BMI from 18.5 to 24.9 kg/m^2^ and the sum of triceps and subscapular skinfolds >90^th^ percentile of the study sample for each sex. ^b^P values calculated by the chi-square test. ^c^Values are mean ± standard deviation. ^d^P values calculated by the Student t-test. Numbers may not add up to total because of missing values.

**Table 3:** Normal weight obesity according to adult life variables, 1978/79 Ribeirão Preto birth cohort.

Variables	Normal weight obesity^a^		P
	No (n = 1,111) n (%)	Yes (n = 111) n (%)	
Age	23.9±0.71^b^	24.0±0.68^b^	0.485^c^
Sex			0.935^d^
Male	496 (90.8)	50 (9.2)	
Female	615 (91.0)	61 (9.0)	
Skin colour			0.013^d^
White	731 (89.5)	86 (10.5)	
Non-white	380 (93.8)	25 (6.2)	
Family income (minimum wages)			0.563^d^
<5	329 (90.1)	36 (9.9)	
5 to 9.9	345 (89.9)	39 (10.1)	
≥10	353 (92.4)	29 (7.6)	
Missing	84 (92.3)	7 (7.7)	
Schooling (years)			0.303^d^
≤8	152 (92.1)	13 (7.9)	
9 to 11	551 (91.8)	49 (8.2)	
≥12	408 (89.3)	49 (10.7)	
Marital Status			0.307^d^
Single	792 (91.5)	74 (8.5)	
Cohabiting	319 (89.6)	37 (10.4)	
Smoking			0.065^d^
No	927 (90.3)	100 (9.7)	
Yes	185 (94.4)	11 (5.6)	
Physical activity			0.010^d^
Sedentary	544 (89.2)	66 (10.8)	
Sufficiently active	220 (89.4)	26 (10.6)	
Active	342 (94.7)	19 (5.3)	
Alcohol consumption (g/day)			0.723^d^
None	296 (90.2)	32 (9.8)	
≤31	595 (90.7)	61 (9.3)	
>31	212 (92.2)	18 (7.8)	
Percentage of fat in the diet	35.8±5.6^b^	37.5±5.4^b^	0.002^c^

a Normal weight obesity defined as a BMI from 18.5 to 24.9 kg/m^2^ and the sum of triceps and subscapular skinfolds >90^th^ percentile of the study sample for each sex. ^b^Values are mean ± standard deviation. ^c^P values calculated by the Student t-test. ^d^P values calculated by the chi-square test.

Subjects with NWO presented higher mean values of BMI, WC, hip circumference, waist to hip ratio, LDL-cholesterol, triglycerides, diastolic blood pressure, subscapular and triceps skinfolds, blood glucose, HOMA2-IR and insulin secretion, and lower values of insulin sensitivity than those without NWO. There were no differences in mean systolic blood pressure. HDL-cholesterol was lower among men with NWO compared to their counterparts but there were no differences among women (Table 4).

**Table 4:** Normal weight obesity according to anthropometric and metabolic parameters, 1978/79 Ribeirão Preto birth cohort.

Variables	Normal weight obesity^a^		P
	No (n = 1,111)	Yes (n = 111)	
Body mass index (kg/m^2^)	21.7±1.7^b^	23.6±1.1^b^	< 0.001^c^
Waist circumference (cm)			
Males	80.4±5.3^b^	87.6±4.9^b^	< 0.001^c^
Females	71.5±4.7^b^	77.2±4.5^b^	< 0.001^c^
Hip circumference (cm)	96.8±4.9^b^	101.1±4.5^b^	< 0.001^c^
Waist to hip ratio (cm)			
Males	0.83±0.04^b^	0.86±0.05^b^	< 0.001^c^
Females	0.74±0.05^b^	0.77±0.05^b^	< 0.001^c^
High density lipoprotein (mg/dL)			
Males	46.2±11.0^b^	42.8±9.8^b^	0.033^c^
Females	54.6±13.1^b^	51.6±12.1^b^	0.092^c^
Low density lipoprotein (mg/dL)	91 (76–110)^d^	104 (88–129)^d^	< 0.001^e^
Triglycerides (mg/dL)	69 (52–95)^d^	89 (64–117)^d^	< 0.001^e^
Systolic blood pressure (mmHg)	115±13^b^	115±13^b^	0.851^c^
Diastolic blood pressure (mmHg)	68±8^b^	70±7^b^	0.008^c^
Subscapular skinfold (mm)	12.7±3.3^b^	20.9±3.5^b^	< 0.001^c^
Triceps skinfold (mm)	11.7±4.6^b^	18.9±4.6^b^	< 0.001^c^
Sum of triceps and subscapular skinfolds (mm)	24.3±6.9^b^	39.8±6.4^b^	< 0.001^c^
Blood glucose (mg/dL)	81 (77–87)^d^	87 (80–91)^d^	< 0.001^e^
HOMA-2 insulin resistance	0.54 (0.36–0.87)^d^	0.77 (0.49–1.22)^d^	< 0.001^e^
HOMA-2 insulin sensitivity	185.7 (115.3–275.5)^d^	129.1 (81.8–203.1)^d^	< 0.001^e^
HOMA-2 β cell function	78.3 (58.0–106.6)^d^	90.9 (65.4–130.1)^d^	0.002^e^

Abbreviations: HOMA, Homeostasis Model Assessment. ^a^ Normal weight obesity defined as a BMI from 18.5 to 24.9 kg/m^2^ and the sum of triceps and subscapular skinfolds >90^th^ percentile of the study sample for each sex. ^b^Values are mean ± standard deviation. ^c^P values calculated by the Student t-test. ^d^Values are median (1st quartile – 3rd quartile). ^e^P values calculated by the Mann-Whitney non-parametric test.

NWO was significantly associated with MS according to the JIS definition (OR = 6.83; 95%CI 2.84-16.47, P<0.001). NWO was also significantly associated with HOMA2-IR (OR = 3.81; 95%CI 1.57-9.28, P = 0.003), low insulin sensitivity (OR = 3.89; 95%CI 2.39-6.33, P<0.001), and high insulin secretion (OR = 2.17; 95%CI 1.24-3.80, P = 0.007). Significant associations between NWO and some components of MS were also detected: high WC (OR = 8.46; 95%CI 5.09-14.04, P<0.001), low HDL-cholesterol (OR = 1.65; 95% 1.11-2.47, P = 0.014) and high triglycerides (OR = 1.93; 95% 1.02-3.64, P = 0.042). Most estimates changed little after adjustment for early and adult life variables: the associations of NWO with low HDL-cholesterol and high triglycerides lost statistical significance and the association of NWO with high blood glucose became statistically significant. After further adjustment for WC, associations of NWO with high blood glucose and high insulin secretion were no longer significant whereas associations of NWO with HOMA2-IR and insulin sensitivity nearly halved but continued to be significant (Table 5).

**Table 5:** Associations between normal weight obesity defined as the sum of the triceps and subscapular skinfolds >90^th^ percentile with metabolic syndrome and its components and insulin resistance, insulin sensitivity and β cell function, 1978/79 Ribeirão Preto birth cohort.

Normal weight obesity	%	Model 1^a^ OR (95% CI)	P ^e^	Model 2^b^ OR (95% CI)	P ^e^	Model 3^c^ OR (95% CI)	P ^e^	Model 4^d^ OR (95% CI)	P ^e^
	**Metabolic Syndrome – JIS^f^**								
No	1.3	1		1		1			
Yes	8.1	6.83 (2.84–16.47)	<0.001	7.22 (2.92–17.85)	<0.001	8.89 (3.32–4.47)	<0.001		
	**High waist circumference^g^**								
No	4.4	1		1		1			
Yes	28.8	8.46 (5.09–14.04)	<0.001	8.37 (5.01–13.99)	<0.001	9.27 (5.32–16.15)	<0.001		
	**Low High Density Lipoprotein^h^**								
No	33.9	1		1		1		1	
Yes	45.0	1.65 (1.11–2.47)	0.014	1.55 (1.02–2.33)	0.038	1.53 (1.00–2.34)	0.053	1.09 (0.69–1.72)	0.721
	**High triglycerides^i^**								
No	6.2	1		1		1		1	
Yes	11.9	1.93 (1.02–3.64)	0.042	1.91 (1.01–3.63)	0.048	1.89 (0.97–3.70)	0.062	1.18 (0.57–2.45)	0.649
	**High blood pressure^j^**								
No	14.6	1		1		1		1	
Yes	16.2	1.11 (0.62–1.97)	0.729	1.19 (0.66–2.13)	0.565	1.17 (0.65–2.13)	0.598	0.87 (0.46–1.67)	0.680
	**High blood glucose^k^**								
No	2.6	1		1		1		1	
Yes	5.5	2.10 (0.84–5.23)	0.110	2.24 (0.89–5.69)	0.089	2.68 (1.01–7.12)	0.048	1.60 (0.54–4.76)	0.395
	**HOMA2- Insulin resistance^l^**								
No	1.9	1		1		1		1	
Yes	6.5	3.81 (1.57–9.28)	0.003	4.01 (1.63–9.87)	0.003	4.91 (1.85–13.04)	0.001	2.94 (1.00–8.68)	0.005
	**Low HOMA2- Insulin sensitivity^m^**								
No	8.4	1		1		1		1	
Yes	26.2	3.89 (2.39–6.33)	<0.001	4.01 (2.45–6.57)	<0.001	4.14 (2.45–6.99)	<0.001	2.22 (1.25–3.96)	0.007
	**High HOMA2- β cell function^n^**								
No	9.4	1		1		1		1	
Yes	16.8	2.17 (1.24–3.80)	0.007	2.48 (1.27–3.97)	0.005	2.26 (1.25–4.08)	0.007	1.58 (0.83–3.00)	0.162

Abbreviations: OR, Odds ratio; CI, Confidence Interval; HOMA, Homeostasis Model Assessment. ^a^adjusted for age, sex and skin color. ^b^adjusted for age, sex, skin colour and early life variables (birth weight, gestational age at birth, maternal schooling, parity, type of delivery and maternal smoking during pregnancy). ^c^adjusted for age, sex, skin colour, early and adult life variables (alcohol consumption, family income, schooling, marital status, smoking, percentage of fat in the diet and physical activity). ^d^adjusted for age, sex, skin colour, early and adult life variables plus WC. ^e^P value calculated by the log-likelihood ratio test. ^f^defined according to the Joint Interim Statement (JIS) of the IDF Task Force on Epidemiology and Prevention, National Heart, Lung and Blood Institute, American Heart Association, World Heart Federation, International Atherosclerosis Society and International Association for the Study of Obesity. ^g^high waist circumference (≥90 cm for men and ≥80 cm for women). ^h^low HDL-cholesterol (<40 mg/dL for men and <50 mg/dL for women). ^i^increased triglycerides (≥150 mg/dL, use of lipid medications or self-reported diagnosis of hypertriglyceridemia). ^j^increased blood pressure (BP) (systolic pressure ≥130 mmHg and/or diastolic pressure ≥85 mmHg, current usage of antihypertensive drugs or previous diagnosis of hypertension). ^k^high fasting blood glucose (≥100 mg/dL), current use of anti-diabetic medication or previously diagnosed diabetes. ^l^Based on the Brazilian Metabolic Syndrome Study - BRAMS criterion (2009) - HOMA2- Insulin resistance >2.8. ^m^HOMA2- Insulin sensitivity was considered low if <90th percentile and normal otherwise. ^n^HOMA2- β cell function was considered high if >90th percentile and normal otherwise.

Table 6 presents models using % BF >23% for men and >30% for women to define NWO. NWO was consistently associated with MS, IR, insulin sensitivity and secretion in all adjusted models.

**Table 6:** Associations between normal weight obesity defined as high percent body fat^a^ with metabolic syndrome and insulin resistance, insulin sensitivity and β cell function, 1978/79 Ribeirão Preto birth cohort.

Normal weight obesity	%	Model 1^b^ OR (95% CI)	P ^e^	Model 2^c^ OR (95% CI)	P ^e^	Model 3^d^ OR (95% CI)	P ^e^
	**Metabolic Syndrome – JIS^f^**						
No	1.4	1		1		1	
Yes	7.5	5.40 (2.21–13.22)	<0.001	5.66 (2.26–14.20)	<0.001	7.36 (2.65–20.45)	<0.001
	**HOMA2- Insulin resistance^g^**						
No	2.0	1		1		1	
Yes	5.8	3.23 (1.26–8.22)	0.014	3.83 (1.32–8.68)	0.011	4.69 (1.65–13.32)	0.004
	**Low HOMA2- Insulin sensitivity^h^**						
No	8.8	1		1		1	
Yes	23.3	3.15 (1.90–5.24)	<0.001	3.28 (1.96–5.47)	<0.001	3.38 (1.96–5.83)	<0.001
	**High HOMA2- β cell function^i^**						
No	9.6	1		1		1	
Yes	14.6	1.79 (0.98–3.25)	0.057	1.89 (1.03–3.45)	0.039	1.85 (1.01–3.49)	0.049

Abbreviations: OR, Odds ratio; CI, Confidence Interval; HOMA, Homeostasis Model Assessment. ^a^high percent body fat>23% for males and >30% for females. Percent body fat was estimated based on the Slaughter's equations using the sum of triceps and subscapular skinfolds. ^b^adjusted for age, sex and skin color. ^c^adjusted for age, sex, skin colour and early life variables (birth weight, gestational age at birth, maternal schooling, parity, type of delivery and maternal smoking during pregnancy). ^d^adjusted for age, sex, skin colour, early and adult life variables (alcohol consumption, family income, schooling, marital status, smoking, percentage of fat in the diet and physical activity). ^e^P value calculated by the log-likelihood ratio test. ^f^defined according to the Joint Interim Statement (JIS) of the IDF Task Force on Epidemiology and Prevention, National Heart, Lung and Blood Institute, American Heart Association, World Heart Federation, International Atherosclerosis Society and International Association for the Study of Obesity. ^g^Based on the Brazilian Metabolic Syndrome Study - BRAMS criterion (2009) - HOMA2- Insulin resistance >2.8. ^h^HOMA2- Insulin sensitivity was considered to be low if <90th percentile and normal otherwise. ^i^HOMA2- β cell function was considered high if >90th percentile and normal otherwise.

## Discussion

In our study, NWO, defined by the combination of excess BF (sum of triceps and subscapular skinfolds >P90 of the study sample) and normal BMI was associated with MS according to the JIS definition (OR = 6.83), HOMA2-IR (OR = 3.81), low insulin sensitivity (OR = 3.89) and high insulin secretion (OR = 2.17) in young adults (23–25 years) from Brazil, a middle-income country. Adjustment for early and adult life variables did not change the estimates appreciably. When we defined NWO as normal BMI and % BF>23% for men and >30 for women results were consistent. Our data suggest that counting only on BMI to identify subjects who are at risk of metabolic disorders later in life may fail to identify an important fraction of the population who, despite having a normal BMI, present excess BF and are also at high risk of metabolic imbalances. It seems that, together with the epidemic of high-BMI obesity [3], there is a normal-BMI obesity epidemic that begins at a young age and is also evident in middle-income countries.

Our study showed that NWO is associated with a high risk of having MS at an early adult age. A 2004 US study also reported that NWO individuals were at increased risk of having MS [21]. A more recent study, carried out in the US using data from the Third National Health and Nutrition Examination Survey (NHANES III), including adults >20 years, showed that NWO was associated with a four-fold increase in the prevalence of MS (16.6% vs. 4.8%) [18]. Our study has some different characteristics compared to that investigation. It is important to note that we measured BF by means of the sum of triceps and subscapular skinfolds, whereas in the American study BF was measured by bioelectrical impedance. Also the criterion for categorization of excess BF differed: we considered those above the sex-specific 90^th^ percentile of the sum of skinfolds as presenting excess BF while in the US study excess BF was defined by the highest sex-specific tertiles of BF percentage. The US study included subjects >20 years old whereas in our study only young adults, aged 23–25 years were included. For the diagnosis of MS the updated NCEP-ATPIII definition was used in the American study [18], whereas in our study we used the new JIS definition. These factors may explain the differences in risk estimates between NWO and MS in the two studies.

We used the JIS definition because it reflects the new emerged consensus to define MS. Furthermore, because our study sample only included young adults more stringent criteria for identification of central obesity would be more appropriate to detect metabolic disorders earlier [44]. Furthermore, the use of lower cut-off points increases the power to detect statistically significant differences in case they exist, while sensitivity increases albeit specificity decreases.

Our study also showed that NWO was associated with an increased risk of presenting high WC (OR = 8.46), high triglycerides (OR = 1.93), and low HDL-cholesterol (OR = 1.65) in young adults. However, no association was observed between NWO and high blood pressure or high blood glucose, although the latter was associated with NWO in the fully adjusted model. The US population-based study, which included a sample of males and females for a total of 6,171 subjects, also showed a significant association between NWO and higher risk of dysregulation of the components of MS (central obesity, high triglycerides, low HDL-cholesterol, high blood pressure and high fasting plasma glucose) [18]. Another study, carried out in Switzerland, which included women only, also showed that NWO was associated with abnormalities in the components of MS [20]. In contrast to the American [18] and the Swiss study [20], our study did not observe an association between NWO and high blood pressure. This could be due to the much younger age of our sample.

Young adults with NWO showed a higher prevalence of IR, as measured by the HOMA-2 model, than those without NWO (OR = 3.81). NWO was also associated with increased IR and low insulin sensitivity. The reduced sensitivity to insulin has been a feature found in subjects with NWO [15], [18], [45]. We also found, in agreement with the American study [18], that increased β cell function was detected among individuals with NWO. Possibly the high insulin secretion is a compensatory response to the reduced insulin sensitivity found in individuals with NWO [46]. Associations between NWO with HOMA2-IR and insulin sensitivity were not totally explained by central obesity because after further adjustment for WC, these associations nearly halved although continued to be significant.

Another recent study, which measured BF with air displacement plethysmography, although not using clinical cut-off points to assess metabolic dysregulation, also reported that non-obese subjects by the BMI criterion but obese by % BF had higher values of WC, blood pressure, triglycerides, glucose, insulin, HOMA and lower values of HDL-cholesterol compared to those with normal BMI and non-obese based on % BF [23].

### Strengths and limitations

We consider the strength of this study to be the fact that it is a population-based cohort study, conducted in a sample of young adults. Adjustment was performed for several adult life variables. In addition, this study incorporates early life factors that have been implicated in the pathogenesis of obesity and IR. These facts allowed us to estimate the association between excess BF and metabolic disorders at an early adult age in subjects with BMI in the normal range in a middle-income country that is undergoing rapid nutrition transition [47]. The narrow age group (from 23 to 25 years of age) shall not be considered a limitation but a particular strength because it eliminates the confounding effect of age and many other age-dependent covariates that may have affected the analysis. Standardized methods were used to assess IR and measurements of body weight, skinfolds, lipids and others.

A potential limitation of our study is related to the method of categorization of excess BF. The sum of subscapular and triceps skinfolds above the sex-specific 90^th^ percentiles of the study sample was used as a proxy for estimating excess BF. It is an arbitrary cut-off, and a definition of NWO different from those used in other studies [18]–[20], [23]. However, results using % BF >23% in men and >30% in women produced consistent results regarding MS and IR. Furthermore, agreement between these two definitions of NWO (>90^th^ of the sum of triceps and subscapular skinfolds and high percent body fat estimated by Slaughter's formula-based equation) was high (kappa = 0.879). Although using three or more measures of skinfold thickness is a validated method to estimate % BF [48], [49], measures of other skinfolds were not available in our database to estimate % BF in adults. We thus estimated % BF using the Slaughter's equations based on the sum of two skinfolds (triceps and subscapular). It is important to note that our sample is composed of young adults aged 23/25 years and the Slaughter's equations were derived for adolescents. We used these equations assuming that % BF would have changed little from 18 to 23/25 years of age. We did not find any other suitable equation to estimate % BF from the sum of triceps and subscapular skinfolds for young adults.

However, the use of subscapular and triceps skinfolds provides a valid indication of excess fat in young people [31]. Selective losses have occurred comparing subjects followed up with those not followed up. Follow-up rates were slightly higher for women, those born preterm, those from better-off families and those whose mothers smoked or were married at the time of the participant's birth. Although statistically significant, these differences were small [32].

### Consequences

These results suggest important questions about the isolated use of BMI to assess obesity. After all, having a normal BMI does not mean no risk for metabolic disorders and consequently for cardiovascular diseases [45]. A BMI cut-off of ≥30 kg/m^2^ has good specificity but misses more than half the people with excess fat [11], [12]. This situation reveals the need for changes in routine clinical evaluation, requiring the incorporation of other simple low-cost measures, like skinfolds or WC, or bioelectrical impedance to evaluate excess BF percentage in individuals with BMI within the normal range. The strong associations found in this study reinforce the need to adopt screening for increased BF as early as possible[13], given that metabolic changes associated with NWO were observed in young adults with normal BMI early in the life course, even in a middle-income country where the burden of obesity-related diseases is not as high as in some developed countries. Although the prevalence rate of MS is low in this young population [50], changes in health care and educational programs, especially encouraging the adoption of a healthy lifestyle, including physical activity and the development of healthy eating habits, are highly advisable in an attempt to halt the spread of future epidemics of obesity in normal-BMI individuals [3], [31].

## Conclusion

In conclusion, our study found associations between NWO and MS and IR early in life in young adults with BMI within the normal range. This implies that using only BMI for the assessment of risk factors for cardiovascular diseases may lead to false-negatives and suggests the need to include the assessment of BF in the routine clinical evaluation of individuals at an early age, even in middle-income countries. Even though prevalence rates of metabolic disorders are low in this population of young adults, as nutrition transition is rapid and as they get older, the burden of obesity-related diseases would tend to be high in the future. Thus, early detection and prevention of this epidemic of normal weight obesity is highly desirable.
